# Relative Age Effects and Gender Differences in the National Test of Numeracy: A Population Study of Norwegian Children

**DOI:** 10.3389/fpsyg.2018.01091

**Published:** 2018-07-02

**Authors:** Tore K. Aune, Rolf P. Ingvaldsen, Ole P. Vestheim, Ottar Bjerkeset, Terje Dalen

**Affiliations:** ^1^Department of Sport Sciences and Physical Education, Nord University, Levanger, Norway; ^2^Faculty of Health Sciences, Nord University, Levanger, Norway

**Keywords:** month of birth, maturation, giftedness, youth development, performance, assessment, school attainment

## Abstract

Relative age effect (RAE) refers to the phenomenon by which children born early in their year of birth perform more highly than children born later in the same cohort. The purpose of this study was to evaluate whether an RAE exists in the Norwegian numeracy test for 5th, 8th, and 9th graders (National sample of 175,760). The results showed that the RAE is consistent across 5th, 8th, and 9th graders for both boys and girls. Mean scores decreased systematically with month of birth for both genders, and the mean scores for boys were higher compared with girls. The most interesting result and novelty is the gender difference in RAE observed analyzing high- vs. low scorers. Boys born early in the year were overrepresented as high scorers (RAE advantage), whereas girls born late in the year were overrepresented as low scorers (RAE disadvantage). It would be beneficial for researchers, teachers and education policymakers to be aware of RAE, both in terms of the practical use and implications of test results and to help identify strategies to adjust for relative age differences in national tests.

## Introduction

Variability and individual differences in human development related to date of birth have interested researchers since the beginning of the twentieth century (Kassel, 1929). Early research revealed that young people who demonstrated eminent performance tended to be born early in their year of birth (Pintner and Forlano, 1934; Huntington, 1938), a phenomenon confirmed by subsequent studies in terms of sports (Musch and Grondin, 2001) and academic achievements (Cobley et al., 2009; Aune et al., 2015).

The relative age effect (RAE) was first demonstrated in the education system, whereby students born late in the school entry-year had a tendency to perform more poorly on end of year attainment than their older counterparts (Jinks, 1964; Freyman, 1965; Armstrong, 1966). Subsequent studies have revealed that RAEs are both systematic and pervasive in the schooling system (Cobley et al., 2009; Aune et al., 2015). Relatively older children tend to score more highly across subject areas, are more often enrolled in gifted and talented programs, and are more likely to represent their school in various sports. Children born later in the year not only achieve lower test scores, but they are also more likely to have special needs and receive special education (Cobley et al., 2009; see also Martin et al., 2004). The relationship between RAE and attainment seems to be consistent across subjects, and early RAEs seem to persist throughout the course of education (Sharp et al., 1994; Massey et al., 1996). As regards physical education and sports, physical maturity might offer older students an advantage, which could be mistaken for superior ability (Aune et al., 2015). Cobley et al. (2008) and Roberts and Fairclough (2012) have identified RAEs among students aged 11–14 years in United Kingdom secondary schools, while Bell et al. (1997) have observed RAEs in ratings of sport performance amongst students aged 16 taking their General Certificate of Secondary Education (GCSE) qualifications in Physical Education.

More alarmingly, however, is the observation that relatively younger students are overrepresented in referral to psychiatric support, and generally display greater health problems (Goodman et al., 2003; Sharp et al., 2009). Perhaps the most disturbing consequence of the RAE was described by Thompson et al. (1999), who found higher incidences of suicide in those born later in the year compared with their earlier born peers within school entry cohorts.

Theoretical support for the existence of the RAE in both sport and the education system rests on the concepts of developmental advantage, greater time for practice, socialization and the Pygmalion effect or self-fulfilling prophecy (Rosenthal and Jacobson, 1968; Harter, 1978). Therefore, RAE in school and sports seems to be enhanced by the fact that an individual will increase performance when expectations of him or her are greater (see Rosenthal, 1987). This is in line with Harter (1978) competence motivation theory, which suggests that children who perceive themselves capable of performing at a high level and who think that they are talented are more likely to continue perfecting their abilities and invest more time and effort into school and other areas in life, with predictable results. Given the relative age difference in the same cohort, such a developmental advantage can be decisive.

One of the primary mechanisms for RAE is that it appears in areas with a high degree of competition and testing, where the activities are organized by age groups (Musch and Grondin, 2001) and strict testing procedures result in the categorization of individuals belonging to the same cohort. Nevertheless, interest in testing students’ school attainment in several subjects and skills has increased in recent decades, and in 1989 the Organisation for Economic Co-operation and Development (OECD, 1989) concluded that Norway was missing a system to control the outcome of students’ learning in school. Several tests have been developed in recent decades to measure learning outcomes across different nations (Hopfenbeck, 2014). In 2004 national tests were also introduced in Norway as part of the national quality assessment system, and national tests are today performed by 5th, 8th, and 9th graders in reading literacy, English and numeracy. These tests are intended to reflect knowledge concerning different competence objectives in the national curriculum (Mausethagen, 2013). National tests were introduced in large part to assess the degree to which schools manage to develop and increase students’ basic skills according to their aims (The Norwegian Directorate of Education and Training, 2015). In addition, test results should act as feedback to schools and stimulate teaching improvements. Finally, they are intended to help improve students’ individual learning progress.

The size of RAEs in school tests has proven to be inversely correlated with age, as the relative age difference between children diminishes over time, and therefore it is reasonable to assume that RAEs would be more prominent in early grades. This has been shown for cognitive abilities and performance (see Bisanz et al., 1995; Morrison et al., 1995; Musch and Grondin, 2001). However, this picture will become complicated by individual differences in early and late maturation, even among children born at approximately the same time and/or in the same month (Anastasi, 1958), although it is reasonable to expect that a large sample size might adjust for this issue. Gender effects have been highlighted in sports (see Vincent and Glamser, 2006), albeit only amongst boys. In contrast, girls seem to experience an almost opposite effect, as their bodily changes during puberty may be detrimental to performance (Malina, 1996). However, these kinds of gender effects are not reported in any studies on RAEs in other topics like numeracy and mathematics. This is surprising as gender differences are found in coping strategies and emotional responses to several environmental factors in children’s and adolescents healthy and maladaptive development (Eschenbeck et al., 2007; Chaplin and Aldao, 2013).

The relationship between RAE and academic achievement is consistent across subjects, and the early RAEs seem to persist throughout the course of education. Given that numeracy and mathematics are given a very high priority in the Norwegian school system, in the present study we address three objectives and hypothesis: (1) RAEs are present in the national tests in numeracy in Norway, and if so, (2) the RAEs are inversely age-dependent, and (3) there are probably gender differences in RAE.

## Materials and Methods

### Subjects and Setting

Data were collected from The National Directorate of Education and Training, reporting the entire population of 5th, 8th, and 9th graders who participated in the annual national test in numeracy in Norway in 2012. The number of students in the tested population totalled 175,760, subdivided into 56,489 in the 5th grade, 59,828 in the 8th grade and 59,443 in the 9th grade (see **Table 1** for detailed information). In the Norwegian school system children starts at school the year they turn six. Approximately 3.4% of the students were exempted from the test because of special needs, and 0.4% did not participate for other reasons.

**Table 1:** Total sample of Norwegian pupils in the National test in numeracy distributed by birth month.

	January	February	March	April	May	June	July	August	September	October	November	December	Total
Girls	7057	6897	7724	7414	7726	7303	7600	7544	7243	7078	6337	6552	86475
Boys	7280	7023	7781	7750	8184	7480	8021	7739	7624	7199	6567	6637	89285
Total	14337	13920	15505	15164	15910	14783	15621	15283	14867	14277	12904	13189	175760


### Design and Analyses

A national test in numeracy is conducted every year in Norway. The test is performed in the first semester of the 5th, 8th, and 9th grades on a date set by The National Directorate of Education and Training. Students are assigned 90 min to complete the test on computers. The test aims to reflect and reveal several levels of numeracy competence across subjects. The national tests in numeracy is in term of content related to the areas of numbers and algebra, measurement and geometry, statistics and probability.

The information provided by the The National Directorate of Education and Training includes information on month of birth, grade, gender and final score on the national test in numeracy. Students’ age was calculated in terms of age by number of months. In many countries, students’ admission into the school system is determined by a single cut-off date; in Norway this is January 1. As a result, all children born in the same calendar year start in the same class, despite the fact that the school year starts in August and ends in June. The month of birth was for some calculations recoded into four quartiles (January–March, April–….etc.). The numeracy test scale was identical for all three grades, representing a continuum from 0 to 58 points (mean = 29.33, *SD* = 11.35). In order to compare students relative to their own grade, grades were converted into *z*-scores for each academic year. For each subpopulation, the standardized scores were also recoded into quartiles. The 25% highest scores (1st quartile) was defined as top scorers, while 25% lowest scores (4th quartile) was defined as low scorers.

The study was conducted according to the Helsinki Declaration and has been approved by the Norwegian Social Science Data Services (NSD).

### Statistics

The study includes the entire population of the three Norwegian school levels (cohorts) from which data were collected, and contains data on gender, age and test performance. Inferential statistics estimate the probability for generalizing results found in groups of random selections of individuals to the entire population. As this is a population study, inferential statistics do not apply (Tabachnick and Fidell, 1996). Consequently, analysis is restricted to the use of descriptive analyses and the chi-squared test. The chi-squared test examines the probability of a distribution of data within a table, rather than the relation between randomly selected groups and the population. The statistical analyses were performed using SPSS (Version 21.0, SPSS, Inc., Chicago, IL, United States).

## Results

**FIGURE 1:**
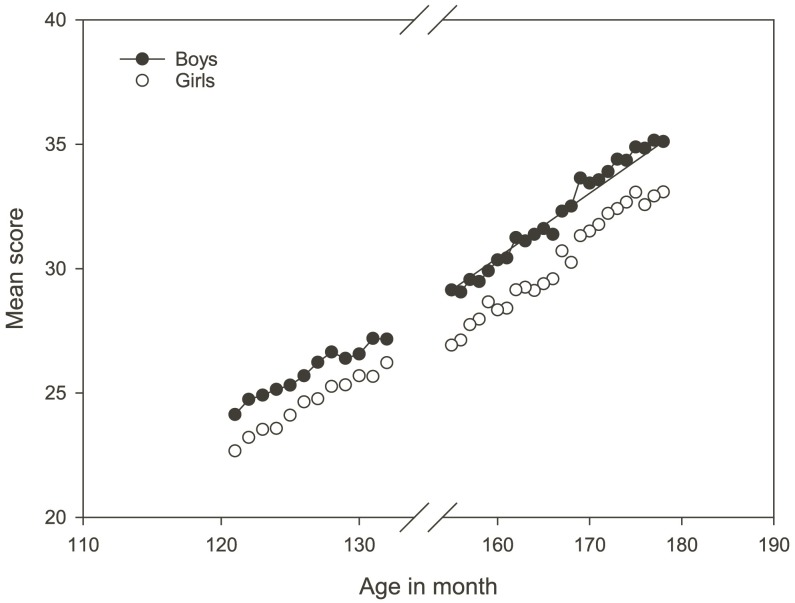
Mean total scores for male and female students in the national test in numeracy related to age measured in months. The figure shows the average score for each month.

The results showed a steady increase in the mean level of scores as a function of age in months for both genders (**Figure 1**). No overlap of test scores between the genders within a confidence interval (CI) set to 95% was identified. The mean scores for grades 5, 8, and 9 were, respectively, 25.22, 29.49, and 33.08. On average for the entire set of population data, boys scored 30.19 and girls 28.44 in the national test in numeracy, a total difference of 1.75. Notably, the gender differences were more pronounced amongst 8th and 9th graders (1.92 and 1.96) compared to 5th graders (1.25).

**FIGURE 2:**
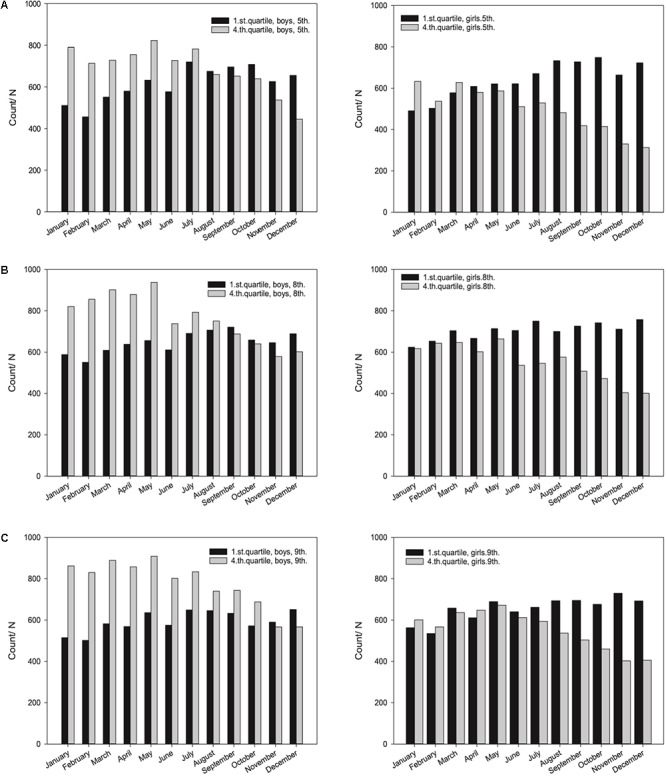
Figure shows top-scorers (1st quartile, gray bars) and low-scorers (4th quartile, black bars) of the national test in numeracy for each month of birth for Norwegian male and female students, respectively, 5th **(A)**, 7th **(B)**, and 8th **(C)** grades.

In order to extent analysis of the association between birth date and scores on the national test in numeracy, the number of males and females as top scorers (25% of the best scores; 1th quartile) and low scorers (25% of lowest scores; 4th quartile) were compared for each grade. **Figure 2** shows that the distribution of both the highest and lowest scores was influenced by date of birth, and it also shows a gender effect: boys born early in the year are clearly overrepresented as high scorers, whereas girls born late in the year are clearly overrepresented as low scorers.

In addition, the students were categorized according to the quarter of the year in which they were born. For the boys born early in the year (January–March), 22% (*n* = 4856) were low scorers in the bottom quartile, whereas 33.5% (*n* = 7391) were in the top scoring quartile. Likewise for the boys born late in the year (October–December), 28.4% (*n* = 5796) were low scorers and 25.8% (*n* = 5264) were high scorers. The corresponding numbers for the girls showed that 24.5% (*n* = 5309) were low scorers, while 25.5% (*n* = 5509) as high scorers for those born in the first quarter of the year. For the girls born in the last quarter, 32.3% (*n* = 6446) were low scorers, and only 18.0% (*n* = 3604) were high scores. These numbers are highly significant, and are presented in greater detail in **Table 2**.

**Table 2:** The scores of the national test in numeracy recoded into quartiles for each subpopulation grade, tabulated against the quarter of birth and split for gender.

		Attainment score			
Gender	Quarter of birth	Low score 0–25% (*N*)	25–50% (*N*)	50–75% (*N*)	High score 75–100% (*N*)
Boys	1	22.0 (4865)	18.6 (4100)	25.9 (5728)	33.5 (7391)
	2	23.4 (5474)	18.8 (4410)	26.1 (6101)	31.7 (7429)
	3	26.2 (6138)	19.8 (4621)	25.6 ( (5981)	28.4 (6644)
	4	28.4 (5796)	20.0 (4086)	25.8 (5257)	25.8 (5264)
Girls	1	24.5 (5309)	22.4 (4850)	27.7 (6010)	25.5 (5509)
	2	26.2 (5878)	22.4 (5020)	27.3 (6133)	24.1 (5412)
	3	28.4 (6359)	23.2 (5203)	27.4 (6130)	21.0 (4695
	4	32.3 (6446)	23.7 (4737)	25.9 (5180)	18.0 (3604)


*The cells show the count of students and row %. Chi-squared analysis was significant for both boys (p < 0.0001, df = 9, Chi = 482.255) and girls (0 < 0.0001, df = 9, Chi = 558.131).*

## Discussion

This large national study of all Norwegian 5th, 8th, and 9th graders confirms a consistent RAE in numeracy testing; the existence of a linear decrease in mean score across months (January through December) for all grades and for both genders. However, the results showed that the distribution of both the highest and lowest scores were not only associated with date of birth, but also had a gender effect. Boys born early in the year are clearly overrepresented as high scorers, whereas girls born late in the year are overrepresented as low scorers.

The systematic association between relative age and mean scores in the national test in numeracy for 5th, 8th, and 9th graders for both genders is consistent with earlier results suggesting that the RAE plays a role in the evaluation of students’ test achievements (Foxman et al., 1990; Sharp et al., 1994; Massey et al., 1996; Bell et al., 1997; Martin et al., 2004;Cobley et al., 2008; Aune et al., 2015). The most obvious explanation for this RAE is that the Norwegian national test in numeracy fails to account for the fact that students are different ages when taking the test. The age at which children start school could contribute to RAEs if younger children find it harder to meet the requirements of a formal curriculum. The greater maturity of older students appears to confer advantages in readiness for intellectual development in the same way as for sports (Bell et al., 1997). Greater maturity might bring psychological advantages too, such as greater confidence, if younger students in comparison come to internalize assumptions and believe themselves to be less capable. The psychological consequences of this advantage may also create greater confidence and self-esteem derived from a comparison of ability to younger, cognitively less mature members of an age group (Cobley et al., 2008). According to Harter (1978) competence motivation theory, students who perceive themselves able to perform at a high level and who regard themselves as talented are more likely to continue perfecting their abilities and invest more time and effort in the subject.

Another explanation for some of the results might be the element of experience. It is suggested that the quantity and quality of practice represent primary mechanisms that explain skill or performance attainment (Baker and Horton, 2004). An 11-month difference in age represents almost a year of opportunities to practice, and hence an opportunity for the oldest students to receive greater academic training. Therefore, it is likely that early performance success also increases motivation.

Furthermore, given that this study has included students aged 10–15, the results indicate that the RAE is a robust phenomenon that is present for academic topics in a relatively wide range of age groups. The existence of discrepancies in physiological maturity related to chronological age differences is well-documented (Bell et al., 1997; Roberts and Fairclough, 2012; Aune et al., 2015), and the present study of numeracy testing indicates that the RAE is also evident for cognitive maturity.

The observed differences in mean scores between boys and girls in numeracy and languages are well-established in previous population studies (Stoet and Geary, 2013). Yet to our knowledge there are no unambiguous psychological, sociological or biological explanations of the gap in performance that typically favor boys in numeracy and girls in languages. There is, however, a persistent claim in several studies that the gender difference is a subtle biased association between sociological rationalizations, and a number of possible sociological reasons for the differences in achievement have been suggested (Else-Quest et al., 2010). The cross-sectional design and variables available in our dataset (gender, age and test result) do not, however, give us any empirical foundation to speculate about the causality of the observed differences. Therefore, future studies should look into more pedagogical dimensions and challenges of these data, complemented by exploration of the conditions and opportunities within the educational system to individualize teaching and other support for students of different ages.

Further analyses demonstrate an interesting gender difference when comparing test score performance of the top scorers versus low scorers for each grade. Boys born early in the year are clearly overrepresented as high scorers (25% highest scores, 1th quartile), whereas girls born late in the year are clearly overrepresented as low scorers (25% lowest scores, 4th quartile). The interpretation of the gender bias could be an example of the Pygmalion effect or Rosenthal effect (Rosenthal and Jacobson, 1968). Indeed, it is a widespread cultural bias to expect boys to be better than girls at logical and theoretical subjects such as mathematics and science (Cunningham et al., 2015). Not taking RAE into account, this bias will be confirmed with positive consequences for the older boys and negative consequences for the younger girls. This is in line with gender differences found in coping strategies and emotional responses to several environmental factors in children’s and adolescents healthy and maladaptive development (Eschenbeck et al., 2007; Chaplin and Aldao, 2013). In a competitive school environment, this may explain the relatively large gender differences observed in the present study.

In turn, the gender-related expectations and beliefs held by parents and other significant persons can be assimilated into their children’s own thoughts of self-competence, appropriateness and the value they attach to subjects. This is likely to affect motivation, which further influences decision-making, behavior and learning effort. These effects can generally be explained by attribution theory and expectancy-valence theory (for more information see Vroom, 1964; Weiner, 1974; Bandura, 1986; Weiner, 2000). Stereotypes regarding female inferiority in mathematics may inhibit girls from achieving and performing to a high level; indeed, girls would probably perform at the same level as boys if assumed gender roles and expectations did not exist and girls could observe female role models excelling in mathematics (Else-Quest et al., 2010). The gender effects found in this study not only confirm a general gender bias, but show that the gender effects are sensitive to RAE.

Academic skills in school might be enhanced by the so-called Pygmalion effect (Rosenthal and Jacobson, 1968; Rosenthal and Babad, 1985), the phenomenon by which an individual performs to a higher standard when expectations of him or her are greater (see Rosenthal, 1987) and vice versa. Research into the Pygmalion effect on classroom achievement has indicated that expectations of a student’s ability trigger a series of verbal and non-verbal interactions that inadvertently influence the student’s subsequent achievement behavior, in what can be termed a self-fulfilling prophecy (Rosenthal and Jacobson, 1968). The combination of gender differences and RAE further complicates this dynamic, as some children will have an advantage or disadvantage due to the way in which teaching is organized in school classes with students born in the same year. All other factors being the same, older boys are likely to demonstrate the greatest ability in numeracy, and young girls the least. This finding is surprising according what is known about the differences in the onset of puberty between boys and girls (Malina, 1996). The teacher’s biased perception of students’ levels of achievement will therefore be confirmed by interacting with the class, unless gender and RAE are not taken into consideration. Such considerations are, however, counterproductive in a competitive environment in which teachers and schools are evaluated based on test results from the national tests. This obviously contributes to the Pygmalion effect and children’s motivation (or lack of) to learn (Carroll, 1992). Initial categorization is also justified as high-attaining children develop more than their low-attaining counterparts. The prophecy thus becomes self-fulfilling (Rosenthal and Babad, 1985). The achieved grade will follow an individual later in life, and may affect his or her possibilities of being admitted to higher education.

A practical implication of the increased interest in testing students’ school attainment in national and transnational tests in several subjects and skills might have a psychologically polarizing effect on students within the same age cohort. Students categorized as gifted gain a positive perceived competence from significant persons as teachers, peers and fellow students, whereas those who attain inferior results in tests are stigmatized.

The negative impact of RAE on childhood school results also seems to systematically affect, albeit to a lesser extent, other areas across the adult life span (Cobley et al., 2009). The immediate damaging effect of the RAE related to school performance may negatively affect children’s motivation and commitment to education, and consequently increase drop-out rates through avoidance behavior (Carroll, 1992; Öhlund and Ericsson, 2001; Goodman et al., 2003). In addition, there exists a growing number of late-born children who are more likely to be identified as learning disabled than older peers (Maddux, 1980), and who are consequently referred for psychological assessments resulting from academic and/or behavioral difficulties. In some cases, these factors might influence individuals’ future level of education, income and health variables (Drabman et al., 1987). Studies has shown that late-born children are overrepresented amongst individuals with lower confidence and self-esteem (Thompson et al., 2004), and in extreme cases also for self-harming and suicidal behavior (Thompson et al., 1999). These consequences may instigate individuals to be predisposed to hopelessness and depression, essential predictive factors of suicidal behavior (Cobley et al., 2009).

Future research should investigate the long-term consequences of the respective advantages and disadvantages of using a prospective design with datasets that include possible confounding factors (e.g., parental socioeconomic status), and evaluate whether advantages result in superior endpoints and vice versa for those experiencing the disadvantages of RAE.

## Conclusion

This study has demonstrated that the RAE is consistent across 5th, 8th, and 9th graders in the Norwegian school system for both boys and girls in terms of numeracy attainment in national tests. The mean scores decreased systematically with month of birth for both genders, and the mean scores for boys were higher than those of girls. Boys born early in the year are overrepresented as high scorers (RAE advantage), whereas girls born late in the year are overrepresented as low scorers (RAE disadvantage). This lends support to previous studies finding gender differences in children’s coping strategies and emotional responses to environmental factors, even though our data cannot pinpoint the specific psychological and social factors behind our findings. Our findings, however, open for further research on gender differences in RAE.

### Practical Implications

The practical implications of such findings are that researchers, teachers, and education policymakers must understand that associations between birth date and performance evaluation can be significant for children. Indeed, repeated and enduring RAEs in several areas affect individuals more than at a single point in life, and it is important to be aware of the potential developmental consequences later in life. Certainly, some of the consequences of the RAE are profound for individuals, and the increased interest and use of national and transnational tests might reinforce these RAEs and the categorization of individuals.

Teachers in Norway are strongly encouraged to practice assessment for learning through formative assessment. Yet, the use of national test results for this purpose might lead to individual ability and skills being interpreted as subject to cognitive maturity via RAE. Therefore, it is critical that teachers recognize the existence of RAE where they use test results to categorize and support individual students’ learning progression.

It would be beneficial for researchers, teachers and education policymakers to be more aware of the RAEs, in order to find strategies to reduce attainment variations due to relative age differences in national tests. An important practical implication of the findings in the present study is to implement knowledge of RAE in teacher education. Interventions that seek to reduce RAEs and their consequences within schools and beyond should be applied and evaluated.

## Author Contributions

All authors listed have made a substantial, direct and intellectual contribution to the work, and approved it for publication.

## Conflict of Interest Statement

The authors declare that the research was conducted in the absence of any commercial or financial relationships that could be construed as a potential conflict of interest.
